# Identification of Important Sugar Binary Mixtures Found in Biorefineries Using Terahertz Time-Domain Spectroscopy

**DOI:** 10.1021/acsomega.5c08490

**Published:** 2025-12-15

**Authors:** Rungroj Jintamethasawat, Pacharamon Somboonsaksri, Nichakarn Termsaithong, Jia-Yi Chia, Sutarat Thongratkaew, Kamonwat Nakason, Thitaphat Ngernsutivorakul, Kantapong Sucharitpongpan, Pongtanawat Khemthong, Nantarat Srisuai, Paramin Sangwongngam, Kamonchanok Duangkanya, Patharakorn Rattanawan, Pakpoom Buabthong, Noppadon Nuntawong

**Affiliations:** 1 National Electronics and Computer Technology Center, 61191 National Science and Technology Development Agency, Pathum Thani 12120, Thailand; 2 School of Integrated Science and Innovation (ISI), Sirindhorn International Institute of Technology, Thammasat University, Pathum Thani 12120, Thailand; 3 National Nanotechnology Center, National Science and Technology Development Agency, Pathum Thani 12120, Thailand; 4 Department of Sanitary Engineering, Faculty of Public Health, 68020 Mahidol University, Bangkok 10400, Thailand; 5 Department of Chemistry, Faculty of Science, 426949 Kasetsart University, Bangkok 10900, Thailand; 6 Department of Science and Technology, 65140 Nakhon Ratchasima Rajabhat University, Nakhon Ratchasima 30000, Thailand

## Abstract

Terahertz (THz) spectroscopy has shown great promise in identifying and quantifying biomolecules whose vibrational modes fall within the terahertz frequency range. This work aims to develop a framework for determining sugar compositions in common chemical reactions that convert C5 and C6 sugars to higher-value products. To simulate these reaction environments, we prepared three types of solid binary mixtures as pellets: glucose-sorbitol, xylose-xylitol, and glucose-fructose, all with varying concentration ratios. Using a terahertz time-domain spectroscopy (THz-TDS) system, we acquired THz spectra of those binary mixture pellets and implemented four types of linear and nonlinear machine learning models to predict sugar compositions from the acquired spectra. Prediction results from test data sets suggest that support vector regression (SVR) shows superior performance over the rest of machine learning models in all binary mixture experiments, with the average and best root-mean-square errors (RMSE) of 5.42 and 2.83% w/w, respectively. Additionally, we investigated the capability of THz spectroscopy to differentiate molecular isomer structures by preparing sample pellets of pure d-xylose and l-xylose. Our results reveal that THz spectroscopy poses challenges in classifying enantiomerism (d-xylose and l-xylose pairs) but still shows potential for identifying functional isomerism (glucose and fructose pairs). These findings demonstrate that THz spectroscopy, combined with optimized machine learning models, offers a promising alternative to gold-standard techniques by enabling simple, rapid, and nondestructive monitoring of chemical compositions during the sugar synthesis.

## Graphical abstract

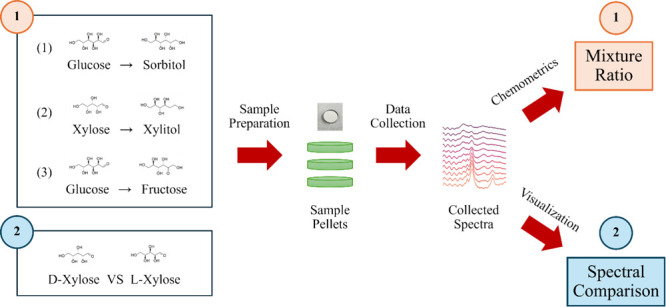

## 1 Introduction

Conversions of C5 and C6 sugars to higher-value sugars serve important roles in various sectors, including the biochemical, food, and medical industries, as they help increase utilization and economic values of biomass wastes. Traditionally, high-performance liquid chromatography (HPLC) has been used for identification of sugar conversion pathways by monitoring yielded sugars and remaining sugar reactants. While HPLC has been regarded as a gold-standard method with high accuracy and chemical specificity, it also poses several challenges.^1,2^ Without adequate expertise, it can be difficult to optimize analytical conditions. Moreover, the method often requires a long analysis time and large volume of solvents.

In contrast, optical spectroscopy techniques offer rapid, simple, and cost-effective measurements compared with chromatography-based techniques. Over the past few decades, several optical spectroscopy techniques have been proposed, and their advantages have been demonstrated in the biochemical, food, and medical industries. Notably, emerging techniques include Raman spectroscopy,^3–6^ near-infrared spectroscopy,^7–10^ and Fourier transformed infrared spectroscopy (FTIR).^11–14^ For instance, Ewanick et al.^15,16^ and Iversen and Ahring^17^ reported the use of Raman spectroscopy specifically for biorefinery applications, while Gillgren and Gorzsás^18^ demonstrated a similar approach using FTIR spectroscopy. It is evident in existing works that those spectroscopy techniques could achieve prediction errors of approximately 15% on real samples.^19,20^ This suggests that spectroscopy techniques may be utilized as alternatives to HPLC analysis, whose accuracy is typically within 3–4%, for screening measurements.^21^ Despite their advantages, Raman spectroscopy has shown limitations in performing quantitative analysis^19,22,23^ as the scattering nature of Raman signals is highly sensitive to environmental and instrumental factors.^21^ Furthermore, absorption-based techniques in the near- or mid-infrared regions often involve a trade-off between high sensitivity and simplicity in sample preparation.^24,25^

Terahertz spectroscopy, which utilizes electromagnetic waves at a frequency range between 0.1 and 10 THz (1 THz = 10^12^ Hz), has received increasing attention in recent years. This is due to its ability to penetrate through nonpolar materials and to identify and quantify biomolecules via unique intermolecular vibrational modes.^26–28^ THz spectroscopy has also demonstrated its abilities to differentiate between certain types of isomers, as shown in refs 29–31 . Such advantages along with recent development of THz devices^32–34^ have made THz spectroscopy a promising tool for a wide range of applications, including food and agriculture,^35^ mineral characterization,^36,37^ and others related to nondestructive diagnostic, inspection, and quality control.^26–28,38–41^ In most circumstances, THz spectroscopy has been utilized for identifying chemical types or compositions. For example,^42–44^ recently proposed techniques for verifying conformation of specific drugs at the molecular level through THz spectra of cocrystals, parent drugs, and their mixtures. Additionally, chemometrics could be employed to analyze complex spectral data in order to determine compositions in the mixtures. This has been shown in our previous work, where THz spectroscopy combined with chemometrics can achieve simultaneous prediction of ternary mixtures representing determinants of coffee.^21^ Similar topics have been explored in previous studies.^38,40,45,46^

This work extends the idea of our previous work^21^ to the context of biorefinery processes by exploiting THz spectroscopy and chemometrics for identifying economically important sugar molecules. In this work, compositions of three solid binary sugar mixtures, which were glucose-sorbitol, xylose-xylitol, and glucose-fructose, were determined from the collected THz spectra using different types of machine learning models. Mixtures used in this study were specifically designed to mimic real-world sugar synthesis reactions, particularly those involving the conversion of C5 and C6 sugars into high-value products.^47–49^ Generalizability of those models, which were trained on binary mixture data sets only, was also investigated by using them to determine the composition of the glucose-xylose-sorbitol ternary mixture. Furthermore, this study evaluates the potential of THz spectroscopy in discriminating functional isomers (d-glucose vs d-fructose) and enantiomers (d-xylose and l-xylose), which, to our best knowledge, have not been explored in existing works.^45,46^

This review is organized as follows: Section 2 describes sample preparation, data acquisition, data processing, and development of the machine learning framework. Section 3 presents the performance evaluation of different types of sugars and provides relevant discussions. Section 4 provides concluding remarks and future directions.

## 2 Materials and Methods

### 2.1 Sample Preparation

Three types of binary mixtures, glucose-sorbitol, xylose-xylitol, and glucose-fructose, were prepared to simulate compositions of sugars found in three reactions: conversion of glucose to sorbitol, conversion of xylose to xylitol, and conversion between glucose and fructose. Glucose, fructose, and xylose in those mixtures had dextrorotatory (*d*) configuration, as shown in Figure 1. For each case, mixtures were prepared as 100 mg solid sample pellets using analytical grade glucose, fructose, xylose, sorbitol, and xylitol with a purity greater than 98%. Xylose and xylitol were purchased from Acros Organics BV, Belgium. Glucose and fructose were purchased from Sigma-Aldrich, United States. Sorbitol was purchased from Tokyo Chemical Industry, Japan. The first set of sample pellets, used for training machine learning models, was prepared in mixture ratios of 0:100, 10:90, 20:80, ..., 90:10, and 100:0 mg (w/w). Three sample pellets were prepared for each mixture ratio, resulting in 33 sample pellets for the training data set. The second set of sample pellets, used for evaluating performance of machine learning models, was prepared in mixture ratios of 5:95, 25:75, 75:25, and 95:5 mg (w/w). Four sample pellets were prepared for each mixture ratio, so there were 16 sample pellets for the test data set.

**Figure 1:**
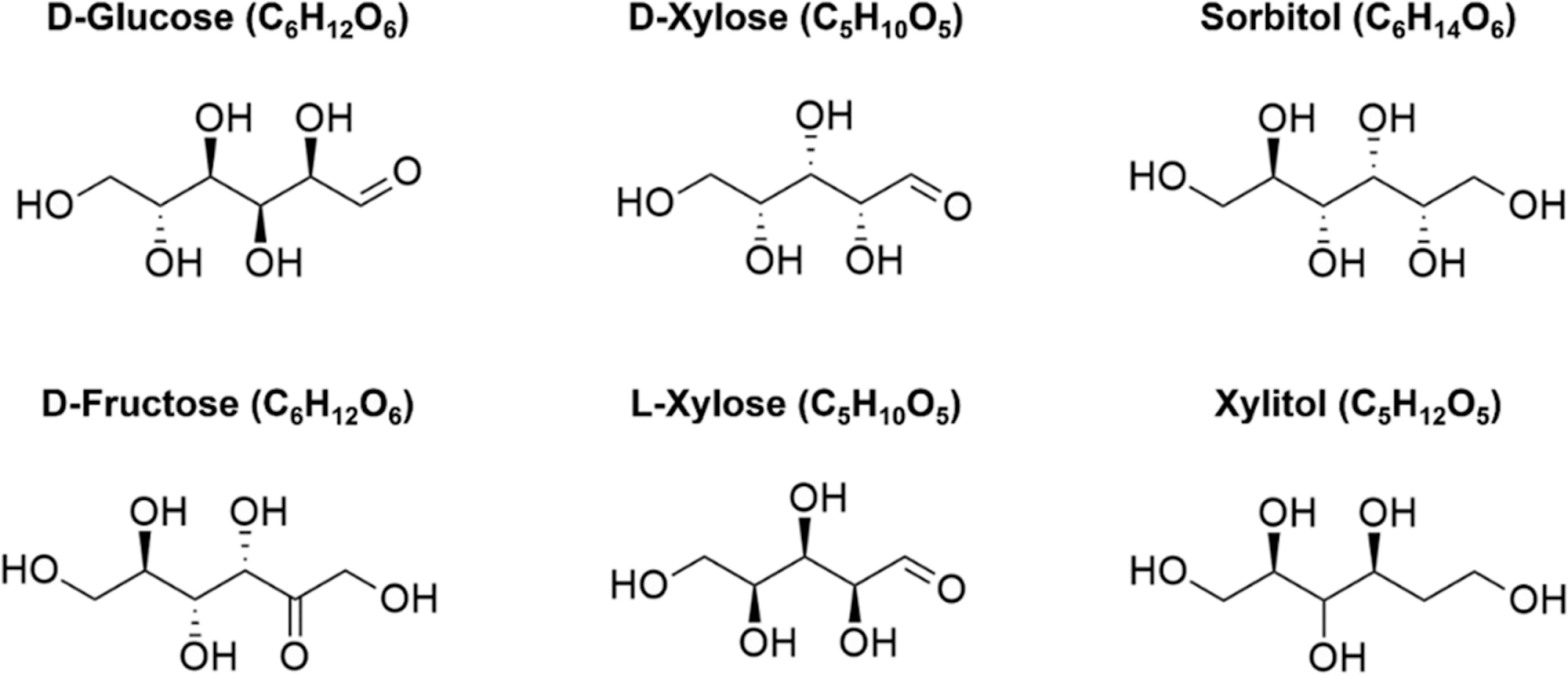
List of sugar molecules studied in this work and their structures.

In addition, 100 mg solid sample pellets of pure d-xylose and l-xylose were prepared. Both d-xylose and l-xylose were analytical grade chemicals with a purity greater than 98% and were purchased from two different sources (Acros Organics BV, Belgium and Thermo Scientific Chemicals, United States). Two sample pellets were prepared for each isomer type and source, resulting in a total of eight sample pellets.

All sample pellets were prepared according to the procedures illustrated in Figure S1a of the Supporting Information. Specifically, preparation steps consisted of first mixing one or more chemicals to the correct weight ratio and then grinding them using a mortar to ensure greatest homogeneity (step 1). After that, they were made into the form of pellets via hydraulic press, which applied 7 ton pressure onto each sample in the circular mold for 5 min (steps 2 and 3). The resulting pellets would have a diameter of 13 mm (step 4), which fit into holes in an acrylic pellet holder mounted onto the THz spectroscopy system. It was possible that different physical properties of sugar chemicals contributed to variations in density, thickness, void fraction, and surface roughness that potentially caused errors in measured absorption spectra. Therefore, to minimize variations of those factors, the total weight, diameter, compaction pressure, and compaction time were controlled to be the same for all of the sample pellets.

### 2.2 Data Acquisition

THz signals were acquired using a THz time-domain spectroscopy (THz-TDS) system (TOPTICA Photonics AG model, Munich, Germany), whose principles and experimental configurations are given in Section S1. During experiments, several sample pellets were inserted into an array sample holder (see Figure S1a) so that THz signals of those samples could be acquired within a single experimental set. Therefore, to ensure sufficient variability in the collected THz signals, each experiment included samples of all concentrations or types. Movement of the sample holder was automated to allow THz signals to be acquired at a given sample pellet and location. The THz-TDS system was operated at room temperature throughout the experiments, and spectral acquisitions were performed within the nitrogen chamber that maintained a relative humidity below 12% RH.

For binary mixtures, THz signals were acquired at 10 uniformly spaced locations per sample pellet. For each type of the binary mixture, three experimental sets were carried out to collect training data set, and another six sets were performed to collect test data set. For d-xylose and l-xylose, as their pellets were made from chemicals bought from two different suppliers, signals were acquired at five uniformly spaced locations per pellet instead. To minimize variability from external disturbances and isolate the isomer type as the only varying factor, all measurements were conducted within the same experiment. Note that, additionally, four reference THz signals, obtained without any sample pellets, were also collected to facilitate data preprocessing described in Section S2. Preprocessed spectra from different binary mixture conditions and isomer types were displayed, and their main features were identified and compared. Refer to Section 3.1 for the resulting spectra and relevant analysis.

### 2.3 Chemometrics

For binary mixture experiments, machine learning technique was additionally employed to predict two compositions in binary mixtures from complex preprocessed spectra. Details about model training, evaluation, and identification of important features are given in the following subsections. Performance of chemometrics was investigated over two choices of spectral frequency ranges: 0.5–2.5 and 1.0–2.5 THz. The implementation of chemometric methods, consisting of model training, model evaluation, and feature importance analysis, was facilitated by packages in the scikit-learn module, which is available in the Python programming language.

#### 2.3.1 Model Training

Supervised models were trained for predicting two compositions in binary mixtures. The following supervised models were chosen: partial least squares (PLS) as a linear model and kernel ridge regression (KRR), support vector regression (SVR), and *k*-nearest neighbor regression (KNR) as nonlinear models. Their details are given in Table 1. Performance of those nonlinear models was investigated and compared against the PLS model, which has been shown to be effective in several prior works.^30,37,39,40^

**Table 1:** List of Machine Learning Models Explored in This Work Along with Their Hyperparameters That Are Tuned during Model Training^a^

ML model	description	tuned hyperparameters
partial least squares (PLS)	finds lower-dimensional latent variables that maximize the covariance between a given spectrum and corresponding mixture ratios.	number of components (3–41)
*k*-nearest neighbors regression (KNR)	predicts the target value by averaging the values of the *k*-nearest neighbors in the feature space.	number of neighbors (2–8), weight (uniform, distance), distance metric (Minkowski, Euclidean, Manhattan)
kernel ridge regression (KRR)	based on ridge regression, but modifies the kernel function to better handle nonlinearity.	regularization parameter (0.00001–5)
support vector regression (SVR)	attempts to fit a function within a specified margin and penalizes deviations outside this margin.	regularization parameter (0.00001–50), kernel (linear, polynomial, radial basis, sigmoid)

a Refer to https://scikit-learn.org/stable for detailed documentation.

Furthermore, while training each model choice, different sets of model hyperparameters were evaluated over given ranges using the leave-one-out cross-validation (LOOCV) technique, and the ones resulting in the lowest prediction error were used in the final model. For a given set of hyperparameters, LOOCV was performed by keeping measurements of the first sample pellet as training data and using the rests to train the model. The excluded measurements were used for evaluating the trained model, and the prediction error was calculated and recorded. This process was repeated until measurements of all of the samples were evaluated. The final prediction error of the model was calculated as the average of all of the prediction errors recorded earlier. The prediction error was calculated as root-mean-square error (RMSE), whose details and equation are given in Section 2.3.2.

#### 2.3.2 Model Evaluation

Test data sets, as detailed in Section 2.1, were used as metrics for evaluating the performance of different machine learning models. Root-mean-square error (RMSE) was calculated for binary mixture experiments using eq 1:

$$\mathrm{RMSE}=\sqrt{\sum_{i=1}^{N}\frac{{∥y_{i}-{ŷ}_{i}∥}^{2}}{N}}$$ 1

where *y*_*i*_, *ŷ*_*i*_, and *N* are predicted concentrations, measured concentrations, and the number of test spectra, respectively.

#### 2.3.3 Feature Importance Analysis

In addition to model training and evaluation, it is worthwhile to examine the behaviors of machine learning models in placing different emphases on various spectral features. This helped us in gauging which frequency range in the spectra was important in order to establish better relationship between chemical and machine learning aspects. Feature importance analysis for each spectral point was performed on the training set by evaluating the degree to which RMSE was affected when the intensity at that spectral point was changed. Specifically, the greater change in RMSE indicated the greater importance of the intensity at that spectral point.

The first 20 frequencies with the highest importances will be marked on the representative spectrum—obtained as an average of all spectra measured from binary mixtures with equal compositions (50:50 mg (w/w)).

## 3 Results and Discussion

### 3.1 Measured THz Spectra

Figure 2 compares the averages of postprocessed THz spectra under different mixture conditions, obtained from three different types of binary mixtures. The average spectrum for each concentration ratio was calculated using 30 spectra in the training data set that were collected from three sample pellets with that concentration ratio. Small shading region accompanying each spectrum represents 95% chance that measurements would lie within that region, indicating that spectral features do not drift significantly when the chamber temperature varies by ±2 °C. Refer to Section S5 for temperature logging results. Furthermore, repeated measurements of pure sample pellets (at least 3 times), shown in Section S3, appear to be consistent across several measurements.

**Figure 2:**
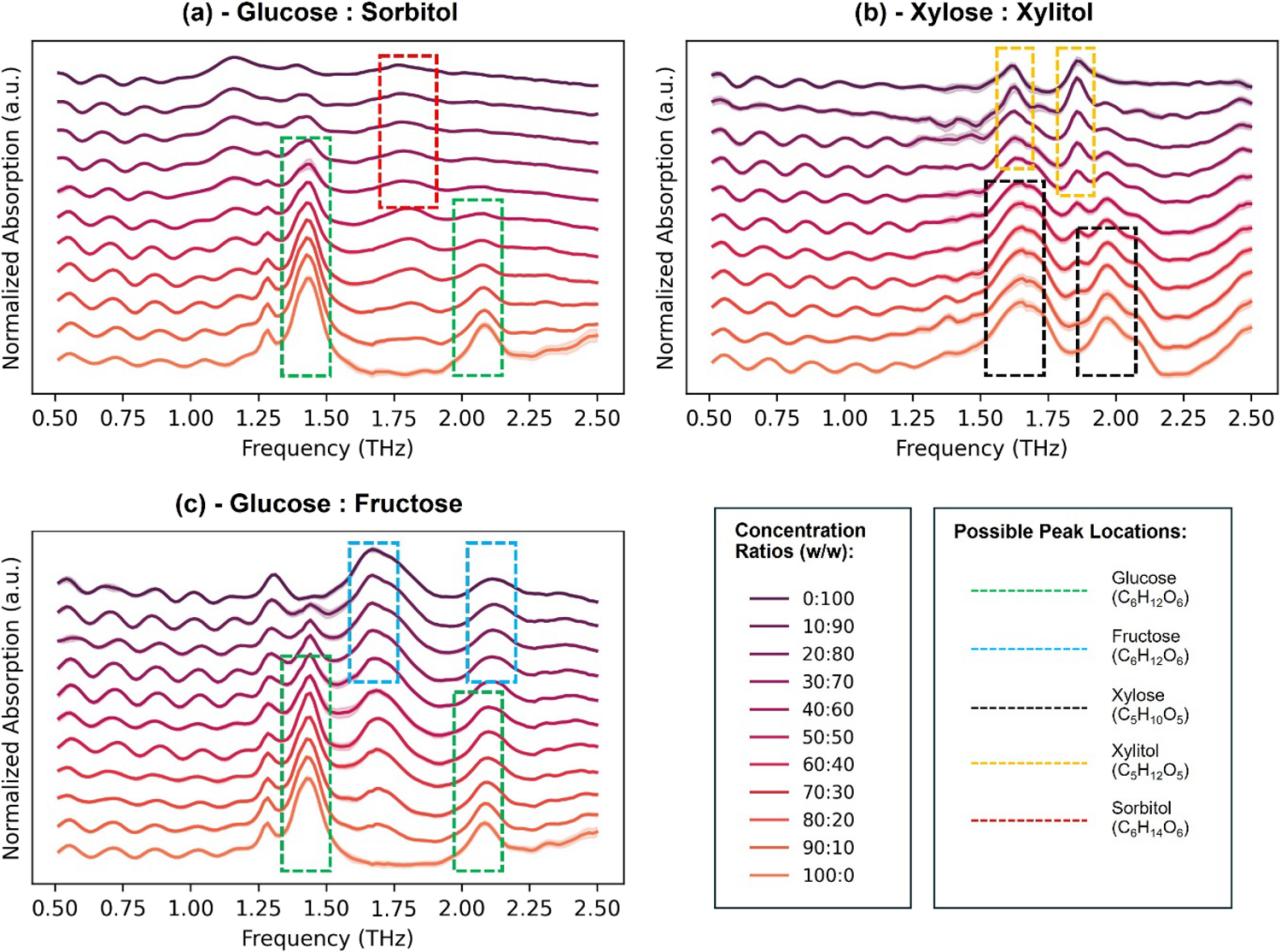
(a) Average postprocessed THz spectra (*n* = 30) obtained under different mixture conditions and shown for (a) glucose and sorbitol, (b) xylose and xylitol, and (c) glucose and fructose mixtures. Important peaks are also highlighted as rectangular overlays on the spectra.

It can be seen from Figure 2 that peaks of several sugar chemicals can be identified from the spectra, and their appearances (especially height) are affected by changes in the concentration ratio. Absorption peaks of glucose are evident at approximately 1.42 and 2.05 THz, consistent with those found in previous studies.^38,45^ Specifically, theoretical calculations and experiments described in ref 38 identified that absorption peaks at 1.42 and 2.05 THz might be associated with rotations of glucose molecules in the macroscopic scale and twisting of the CH_2_OH functional group, respectively. Meanwhile, absorption peaks of fructose can be observed at approximately 1.69 and 2.12 THz. This is confirmed by ref 38, which also states that those absorption peaks might be associated with translational motion and deformation of the carbon ring of fructose molecules, respectively. For xylose (d-xylose), absorption peaks are found at approximately 1.67 and 1.96 THz,^50^ corresponding to intramolecular vibrations being likely due to hydrogen bond stretching. Xylitol could be identified via peaks located approximately at 1.62 and 1.87 THz.^50^ Again, these might arise due to intramolecular vibrations, such as structural deformation and hydrogen bond stretching. However, to the best of our knowledge, there have been no studies that explicitly aim to identify sorbitol peaks in the THz range. Still, in Figure 2a, there seems to be subtle changes in spectral intensities at around 1.80 THz. This suggests that spectral intensities at that location might be affected by the presence of sorbitol, but note that theoretical calculations and experiments should be performed in the future to validate this assumption. Those features of all of those peaks are crucial for identifying and differentiating sugar compositions in binary mixtures. However, quantifying concentrations of individual sugar types by only considering their peak intensities (as performed in Section S4) can be challenging and results in high limit of detection (LOD) and high limit of quantification (LOQ). Results in Section 3.2 would demonstrate how machine learning could be utilized to overcome such limitations.

Furthermore, distinguishable peaks of glucose and fructose, as observed in Figure 2c, suggest the potential of THz spectroscopy to identify and quantify functional isomers. While this seems interesting at first glance, differentiating enantiomers—a special type of isomerism—could be much more challenging even in the THz frequency range. This is illustrated by averages of THz spectra of d-xylose and l-xylose (Figure 3) being nearly identical, as well as THz spectra of other enentiomers reported in refs 30 and 31. Unlike glucose and fructose, which are isomers being different in functional groups, d-xylose and l-xylose possess very similar molecular structures and geometries that are just mirror images of each other, as shown in Figure 1. This might lead to identical intramolecular (rotations, stretching, and twisting) and intermolecular (hydrogen bonding) vibrational modes in their crystalline structures. Also, note that this case is different from a study in ref 29, which employs THz spectroscopy to distinguish between α-d-lactose and β-d-lactose anomers, a specific type of stereoisomerism characterized by detectable variations in the hydroxyl group orientation at the anomeric carbon.

**Figure 3:**
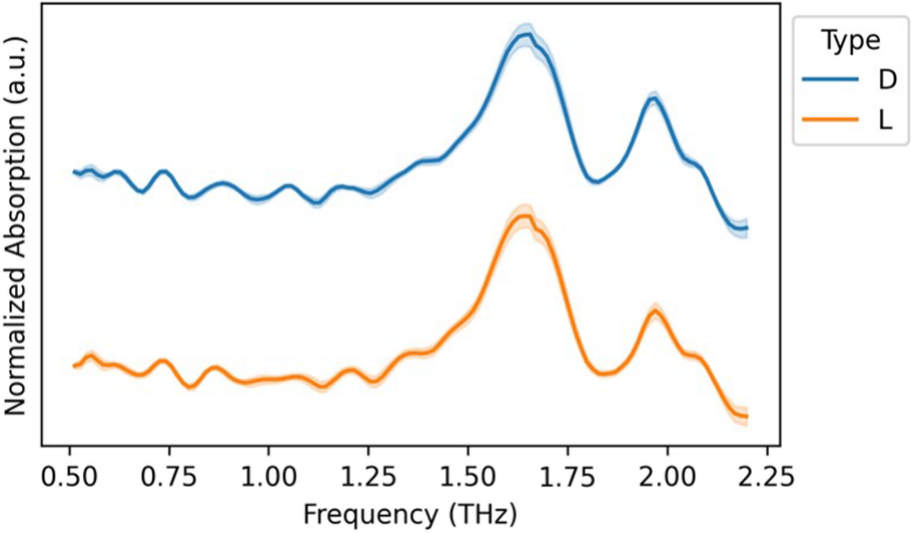
Average postprocessed THz spectra (*n* = 20) of d-xylose and l-xylose.

### 3.2 Predictions of Sugar Compositions in Binary Mixtures

Finding concentrations of single peaks could be Table 2 showing RMSE values of different machine learning models when predicting sugar compositions of three types of binary mixtures. RMSE values were also investigated on both training data sets (by the optimal model obtained during leave-one-out cross-validation) and test data sets over two frequency ranges (0.5–2.5 and 1.0–2.5 THz). According to the table, RMSE values tend to decrease in most cases, for both training and test data sets, when measurements within the narrower frequency range are utilized. This is expected, as the majority of important peaks are located at frequencies above 1.0 THz.

**Table 2:** Performance Benchmark of Different Machine Learning Models, Which Can Be Evaluated through Their RMSE Values

		RMSE (% w/w)			
		0.5–2.5 THz		1.0–2.5 THz	
mixture	ML model	training	test	training	test
glucose & sorbitol	PLS	9.51	6.88	7.68	6.02
	KNR	11.39	4.07	11.15	4.24
	KRR	8.17	7.02	7.32	7.21
	SVR	6.45	5.94	6.17	2.83
xylose & xylitol	PLS	6.97	6.94	7.21	7.40
	KNR	12.13	9.88	11.72	10.93
	KRR	9.63	9.78	9.88	8.18
	SVR	6.95	7.07	6.85	7.11
glucose & fructose	PLS	3.87	7.71	4.40	7.48
	KNR	9.17	5.51	8.88	5.52
	KRR	5.16	8.12	5.16	7.98
	SVR	4.14	7.13	4.18	6.32
average [standard deviation] from 3 binary mixture experiments	PLS	6.79 [2.31]	7.18 [0.38]	6.43 [1.45]	6.97 [0.67]
	KNR	10.89 [1.26]	6.49 [2.47]	10.59 [1.23]	6.90 [2.90]
	KRR	7.65 [1.86]	8.31 [1.13]	7.45 [1.93]	7.79 [0.42]
	SVR	5.85 [1.22]	6.71 [0.55]	5.73 [1.13]	5.42 [1.86]

The choice of the machine learning model also affects the resulting RMSE value. Table 2 shows that the combination of SVR and spectra measured within the narrower frequency range of 1.0–2.5 THz yields the best performance in the test data set, achieving the lowest RMSE in two out of three experiments. SVR also appears to result in the lowest RMSE value on average (5.42 ± 1.86% w/w for the 1.0–2.5 THz frequency range), as seen in the last row of Table 2. This could be attributed to the SVR model in its ability to handle nonlinear effects (if using nonlinear kernels) and outliers or extreme values in the measurements. Therefore, the SVR model appears to have the best overall performance compared to the other three models.

On the other hand, KNR might outperform SVR in some cases during the test phase, such as glucose and sorbitol experiments at the 0.5–2.5 THz frequency range, and glucose and fructose experiments at both 0.5–2.5 and 1.0–2.5 THz frequency ranges. This could be attributed to an excessive degree of nonlinearity in the KNR model. However, it exhibits the poorest performance during the training phase, suggesting a potential risk of model overfitting. Meanwhile, PLS and KRR models tend to show the moderate performance compared to the other two models, where in some case PLS shows slightly superior accuracy compared to SVR, such as xylose and xylitol experiments at 0.5–2.5 THz. However, they are less susceptible to overfitting, as evidenced by the similar RMSE values between their training and test sets. This can be attributed to PLS’s ability to reduce data dimensionality and KRR’s use of regularization technique, both of which help mitigate overfitting.

Note also that in most cases, RMSE values resulting from the prediction of THz spectra in the test data sets appear to be less than those resulting from the prediction of THz spectra in the training data sets. This is somewhat unexpected, but collecting more spectra for both training and test data sets might help in identifying the root cause more precisely.

Comparisons between predicted and actual sugar concentration ratios, for the SVR model, are presented in Figure 4. In this case, only THz spectra obtained within the frequency range of 1.0–2.5 THz are utilized and analyzed. As can be seen from glucose and sorbitol (Figure 4a) and xylose and xylitol (Figure 4b) experiments, predictions from multiple measurements for a given concentration ratio tend to be distributed around the actual value. While this might lead to a high RMSE value, averaging predictions from multiple measurements appears to help in reducing uncertainties and improving accuracy, as evident in Table 3 showing the breakdown of the average predicted concentration and RMSE value. Averaging approach also helps reduce uncertainties even in the case of glucose and fructose experiments (Figure 4c), but slight overestimation and underestimation still occur due to the inherent bias from either measurements or chosen SVR model.

**Figure 4:**
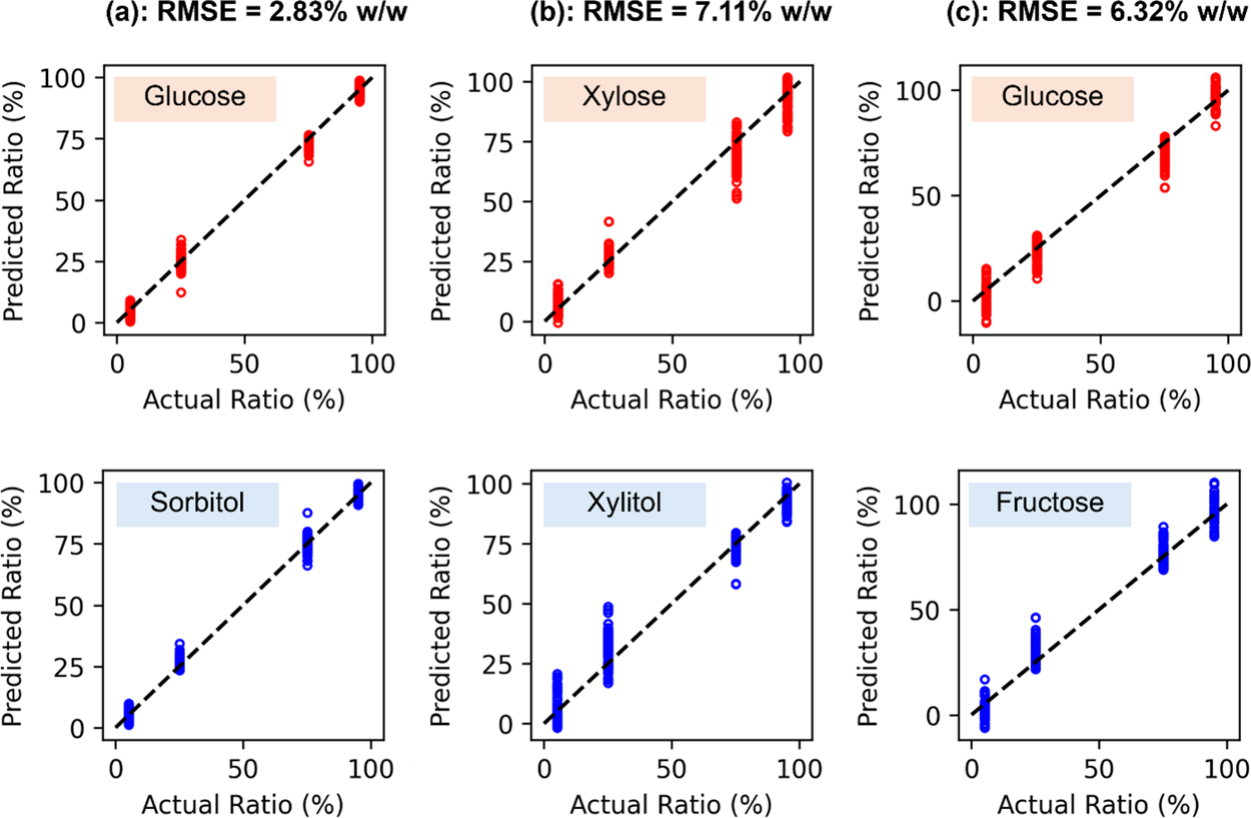
Comparisons between actual sugar concentration ratios and predicted sugar concentration ratios using the SVR model and spectra within the frequency range of 1.0–2.5 THz. Comparisons are made for predictions of (a) glucose and sorbitol, (b) xylose and xylitol, and (c) glucose and fructose mixtures. Their associated RMSE value is also given for each type of the binary mixture.

**Table 3:** Average Predicted Concentration and RMSE Value Determined for Each Specific Concentration Ratio, Obtained as the Results of Using the SVR Model^a^

	% w/w			% w/w	
sugar 1	actual	predicted [RMSE]	sugar 2	actual	predicted [RMSE]
glucose	5	4.66 [2.15]	sorbitol	95	95.34 [2.15]
	25	24.67 [3.69]		75	75.33 [3.69]
	75	73.02 [3.05]		25	26.98 [3.05]
	95	94.63 [2.14]		5	5.37 [2.14]
xylose	5	7.23 [4.22]	xylitol	95	92.77 [4.22]
	25	27.46 [5.02]		75	72.54 [5.02]
	75	67.58 [10.03]		25	32.42 [10.03]
	95	91.00 [7.67]		5	9.00 [7.67]
glucose	5	3.28 [5.96]	fructose	95	96.72 [5.96]
	25	23.19 [5.42]		75	76.81 [5.42]
	75	69.21 [8.60]		25	30.79 [8.60]
	95	96.37 [4.59]		5	3.63 [4.59]

a For each experiment and concentration ratio, those metrics were calculated from 40 test spectra (4 samples per experiment × 10 test spectra per sample) collected within the 1.0–2.5 THz frequency range.

Concerning ecological interpretation, glucose can be almost completely converted to sorbitol through catalytic hydrogenation, achieving yields exceeding 90% as reported in the literature.^51^ The relatively low RMSE values of the SVR model near the edge concentration regions indicate that the model performs well in predicting the conversion of glucose to sorbitol, a process relevant to the sweetener and pharmaceutical industries. Similarly, xylose can be converted to xylitol via catalytic hydrogenation with very high yields (>90%).^52^ Although the RMSE increases when the xylose concentration exceeds that of xylitol, this catalytic reaction is unidirectional and leads to a predominant formation of xylitol upon completion. In contrast, the isomerization of glucose to fructose is typically incomplete and reversible.^53^ The RMSE values remain consistent throughout the prediction range for glucose and fructose mixtures, except for those with approximately 75:25% w/w glucose-to-fructose ratios. This suggests that the SVR model should be capable of quantifying glucose-fructose mixtures across the entire compositional range in general. Apart from that, there could be limitations when using machine learning models trained from binary mixture data sets to predict sugar compositions in systems where two or more chemicals show dominant spectral peaks in the THz frequency range. This was investigated in Section S6, where existing SVR models were used for quantifying compositions of pure glucose, xylose, and sorbitol pellets (with 33:33:34% w/w as the actual glucose-to-xylose-to-sorbitol ratio). Such findings emphasize the practicality in using the proposed analysis and prediction framework in complex circumstances with the presence of several chemicals and greater measurement difficulties.

### 3.3 Feature Importance Analysis

Figure 5 highlights the first 20 spectral points that are considered as the most important for the SVR model, which is chosen due to its best average RMSE in most cases (see Table 2 for mode details). THz spectra shown in all subfigures are representative spectra measured from all three types of binary mixtures with equal compositions (50:50 mg (w/w)) at the frequency range of 0.5–2.5 THz. As seen from the figure, machine learning puts more emphasis on spectral points that are parts of peaks rather than other locations that are less relevant. This is in accordance with the important peak locations identified in Section 3.1. Furthermore, note that there are no important spectral features at frequencies below 1 THz, which explains why prediction performances are not noticeably different for both choices of the frequency range.

**Figure 5:**
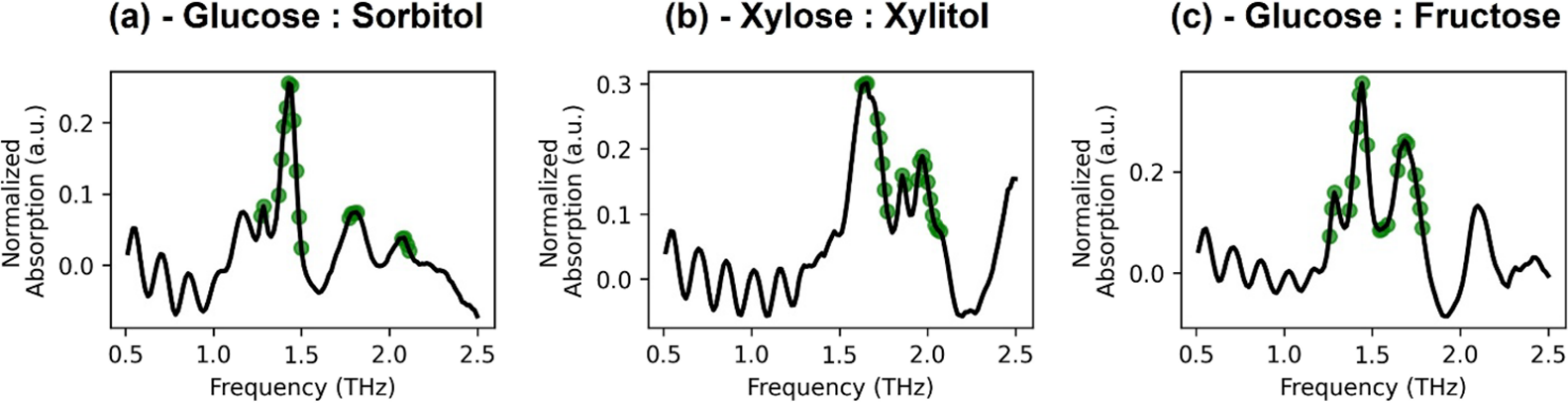
First 20 spectral points that are considered as the most important features for the SVR model, displayed for (a) glucose and sorbitol, (b) xylose and xylitol, and (c) glucose and fructose mixtures.

## 4 Conclusions

This work successfully demonstrated the application of THz-TDS spectroscopy combined with machine learning techniques for determining sugar compositions in binary mixtures relevant to biorefinery processes. Feature importance analysis was also performed to verify the effectiveness of machine learning models in utilizing the characteristic absorption peaks of sugars in the THz range with the most informative spectral features occurring above 1 THz. Furthermore, an investigation into isomerism confirmed that THz spectroscopy can effectively distinguish between functional isomers (glucose and fructose) but has limitations in identifying enantiomers (d-xylose and l-xylose), which exhibit nearly identical THz spectra. This limitation is consistent with the theoretical understanding of the behaviors of enantiomers.

Methods proposed in this work provide a framework for developing rapid and nondestructive analytical techniques for monitoring sugar compositions in biorefinery processes. While the current framework demonstrates promising performance for binary mixtures, future work should focus on extending this approach to more complex mixtures with multiple sugar components, as would be encountered in actual biorefinery reactions. Further improvements could also be achieved by incorporating advanced data preprocessing techniques and exploring deep learning models that might better capture subtle differences.

## Supplementary Material



## Notes

Details about data acquisition system, data preprocessing, and calculation of limit of detection (LOD) and limit of quantification (LOQ) values; THz spectra obtained from repeated measurements of pellets of the same type, temperature logging results, and predictions of sugar compositions in the ternary mixture using machine learning models trained from binary mixture data sets (PDF)
