# Supplementary material for: Identification of Important Sugar Binary Mixtures Found in Biorefineries Using Terahertz Time-Domain Spectroscopy
Source: ACS Omega. 2025 Dec 15;10(51):62872–80. doi: 10.1021/acsomega.5c08490 (PMC12756803; doi:10.1021/acsomega.5c08490)
Supplement: Supplementary file 1 [file ao5c08490_si_001.pdf]

SUPPORTING INFORMATION

Identification of Important Sugar Binary Mixtures Found in Biorefineries Using Terahertz Time-Domain Spectroscopy

Rungroj Jintamethasawat[1], Pacharamon Somboonsaksri[1], Nichakarn Termsaithong[1], Jia-Yi Chia[2], Sutarat Thongratkaew[3], Kamonwat Nakason[4], Thitaphat Ngernsutivorakul[5], Kantapong Sucharitpongpan[1], Pongtanawat Khemthong[3], Nantarat Srisuai[1], Paramin Sangwongngam[1], Kamonchanok Duangkanya[1], Patharakorn Rattanawan[1], Pakpoom Buabthong[6,], Noppadon Nuntawong[1,*]*

[1]National Electronics and Computer Technology Center, National Science and Technology Development Agency, Pathum Thani, Thailand 12120

(Email: noppadon.nuntawong@nectec.or.th)

[2]School of Integrated Science and Innovation (ISI), Sirindhorn International Institute of Technology, Thammasat University, Pathum Thani, Thailand 12120

[3]National Nanotechnology Center, National Science and Technology Development Agency, Pathum Thani, Thailand 12120

[4]Department of Sanitary Engineering, Faculty of Public Health,

Mahidol University, Bangkok, Thailand 10400

[5]Department of Chemistry, Faculty of Science, Kasetsart University,

Bangkok, Thailand 10900

[6]Department of Science and Technology, Nakhon Ratchasima Rajabhat University, Nakhon Ratchasima, Thailand 30000 (Email: pakpoom.b@nrru.ac.th)

S1. Terahertz Time-Domain Spectroscopy System

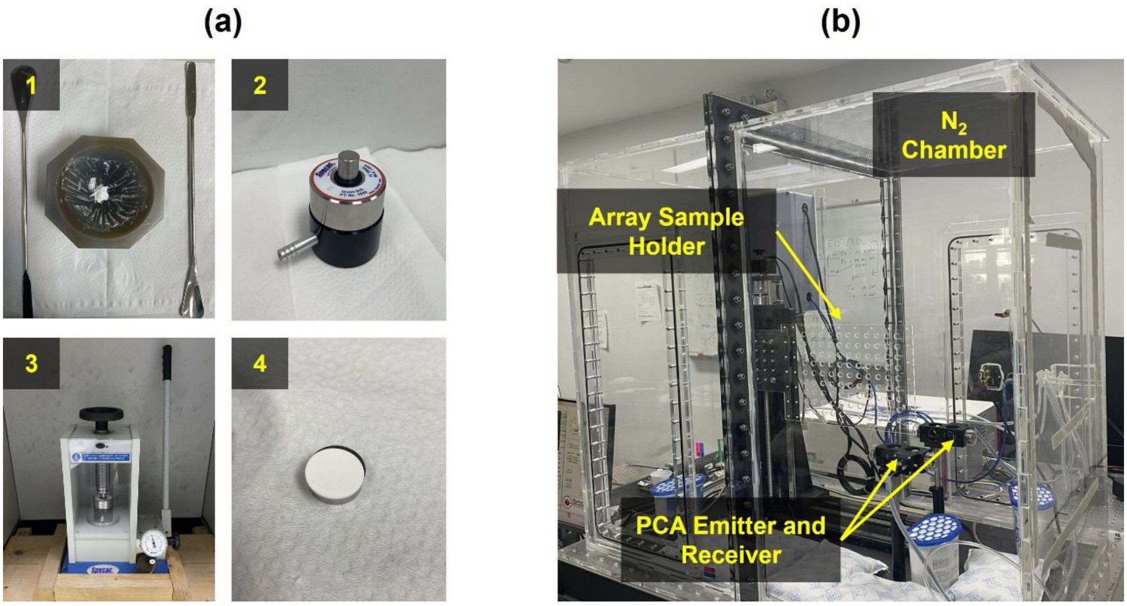

Figure S1. (a) Steps in preparing sample pellets. (b) Experimental setup showing components required for acquisition of THz spectra.

THz time-domain spectroscopy (THz-TDS) system (TOPTICA Photonics AG model, Munich, Germany), shown in Figure S1(b) was utilized to acquire time-domain signals of sample pellets prepared by procedures described in Figure S1(a). The acquired time-domain

signals had corresponding frequency within THz range, and were generated and received by a pair of photoconductive antenna (PCA), one being an emitter and another being a receiver. Specifically, when the emitter was induced by a femtosecond laser at wavelength of 780 nm, it would generate free-carriers, which were subsequently recombined with the holes in the PCA substrate. Such emission and recombination of free carriers generated an electromagnetic pulse. The generated pulse would then be detected by the PCA receiver that was also induced by another beamline from the femtosecond laser. Since signal intensity could be detected at only one time point on the pulse per acquisition, laser beamline to the receiver was delayed for gating the detection at different time points with intervals of 0.05 ps until acquisition of one full pulse was complete. Apart from the acquisition components, both PCA emitters and receivers, and pellet holder, were installed in an airtight chamber. The chamber might be optionally filled with nitrogen to ensure minimal disturbances due to atmospheric moisture.

S2. Data Pre-Processing

After time-domain THz signals were obtained, their corresponding spectra were calculated via fast-Fourier transform (FFT). This was done using NumPy module, which is available in the Python programming language. Then, spectrum of each sample was pre-processed in the following order:

1) Normalization: The sample spectrum was normalized by the average of reference spectra obtained from the same experimental set according to Eq. S1:

$$E = 1 - \frac{\sqrt{E_{sam}^2+\beta^2}}{\sqrt{E_{ref}^2+\beta^2}} \quad \text{(S1)}$$

where E_{ref} , E_{sam}, and E represent reference, sample, and normalized absorption THz spectra, respectively. β = 0.001, was added to both nominator and denominator parts to prevent division by too-small numbers and mitigate undesirable effects due to low signal-to-noise ratio (SNR).

2) Baseline removal: Baselines of normalized spectra, which occurred due to nonlinear scattering effects between the sample and the THz signals, were corrected. This was achieved by applying doubly reweighted penalized least squares (dr-PLS) method. The RamPy module in Python was utilized to implement this baseline removal algorithm.
3) Cropping: Spectral intensities in the low (<0.5 THz) or high (>2.5 THz) frequency range were cropped out as they might exhibit high uncertainty or low SNR. This approach was also used to investigate prediction performance over different choices of spectral frequency ranges.

S3. Evaluation of Robustness in Measurements of Sample Pellets

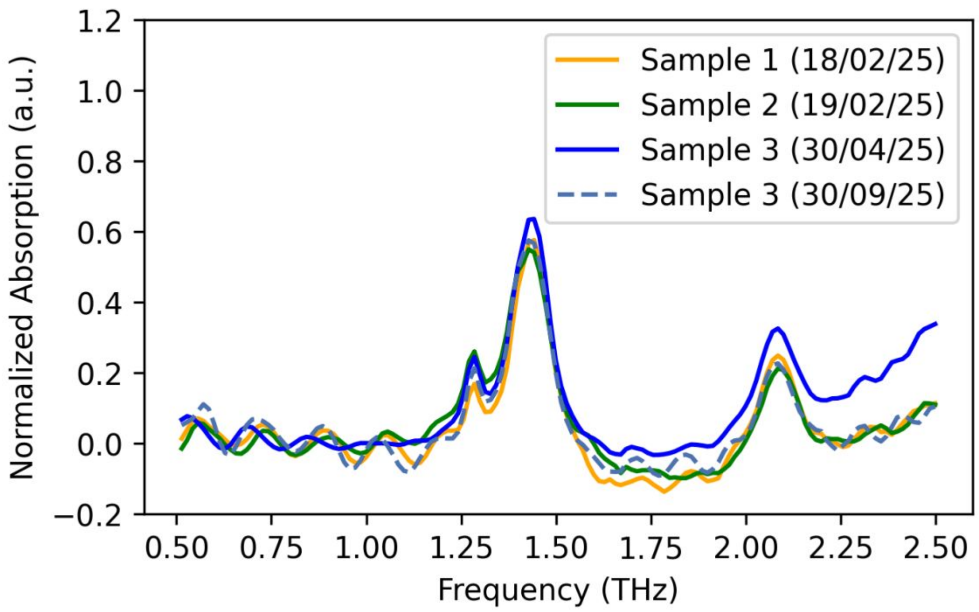

Figure S2. Average post-processed spectra (n = 10) of 3 pure glucose pellets (samples 1-3) collected over 4 different dates. All samples were prepared a day prior to their first spectral measurements. Note that sample 3, measured previously on 30th April 2025, was measured again on 30th September 2025. As can be seen, all average spectra show similar peaks and features in almost entire frequency range, except between 2.25-2.50 THz which might be attributed to misalignments arising when placing the pellet onto the array sample holder. This confirms robustness in spectral measurements.

S4. Limit of Detection (LOD) and Limit of Quantification (LOQ) Calculation

Tables S1-S3 display intensities of spectral peaks corresponding to the presence of each sugar type, recorded at 11 different mixture ratios and for three binary mixture experiments. Dataset in each table was used for constructing two linear calibration functions for two sugar types, where each represents intensity of spectral peak as a function of sugar concentration. Upon performing linear regression to find the calibration function, both slope estimate, i.e., intensity divided by concentration (a.u./% w/w), and its corresponding standard deviation (a.u.) were

also calculated. Consequently, LOD ($3\sigma_{blank}$) and LOQ ($10\sigma_{blank}$) for each binary mixture experiment can be obtained by the following Eqs. S2 and S3:

$$LOD = \frac{3\sigma_{blank}}{Slope} \tag{S2}$$

$$LOQ = \frac{10\sigma_{blank}}{Slope} \tag{S3}$$

where σ_{blank} is the standard deviation of the blank-pellet measurements, which can be estimated as standard deviation of the calculated slope.

Table S1. Intensities (heights) of spectral peaks corresponding to glucose and sorbitol, recorded at 11 different concentrations. For each sugar type, a peak was identified within the frequency range provided under its corresponding sugar name. Calculated LOD, LOQ, and other intermediate quantities (slope and standard deviation) are also given at the bottom of the table.

Concentration (% w/w)	Peak Intensity - Glucose (Within 1.3-1.5 THz)	Peak Intensity - Sorbitol (Within 1.7-1.9 THz)
0	0.073	-0.057
10	0.068	-0.029
20	0.116	0.017
30	0.149	0.054
40	0.182	0.064
50	0.255	0.074
60	0.313	0.066
70	0.394	0.075
80	0.469	0.077
90	0.551	0.075
100	0.585	0.074
**Slope**	0.006	0.001
**Standard error**	0.036	0.027
**LOD (% w/w)**	19.060	70.107
**LOQ (% w/w)**	63.532	233.691

Table S2. Intensities (heights) of spectral peaks corresponding to xylose and xylitol, recorded at 11 different concentrations. For each sugar type, a peak was identified within the frequency range provided under its corresponding sugar name. Calculated LOD, LOQ, and other intermediate quantities (slope and standard deviation) are also given at the bottom of the table.

**Concentration (% w/w)**	**Peak Intensity - Xylose (Within 1.5-1.7 THz)**	**Peak Intensity - Xylitol (Within 1.8-1.9 THz)**
0	0.140	0.149
10	0.191	0.124
20	0.216	0.135
30	0.225	0.143
40	0.278	0.165
50	0.301	0.159
60	0.356	0.187
70	0.386	0.189
80	0.421	0.213
90	0.419	0.255
100	0.449	0.177
**Slope**	0.003	0.001
**Standard error**	0.015	0.024
**LOD (% w/w)**	14.130	77.514
**LOQ (% w/w)**	47.100	258.380

Table S3. Intensities (heights) of spectral peaks corresponding to glucose and fructose, recorded at 11 different concentrations. For each sugar type, a peak was identified within the frequency range provided under its corresponding sugar name. Calculated LOD, LOQ, and other intermediate quantities (slope and standard deviation) are also given at the bottom of the table.

Concentration (% w/w)	Peak Intensity - Glucose (Within 1.3-1.5 THz)	Peak Intensity - Fructose (Within 1.6-1.8 THz)
0	0.156	-0.049
10	0.151	0.027
20	0.205	0.101
30	0.286	0.205
40	0.340	0.243
50	0.375	0.261
60	0.440	0.308
70	0.453	0.325
80	0.485	0.337
90	0.545	0.353
100	0.580	0.352
**Slope**	0.005	0.004
**Standard error**	0.021	0.051
**LOD (% w/w)**	13.883	39.285
**LOQ (% w/w)**	46.275	130.950

S5. Temperature Logging Results

Table S4. Temperature logging results over the duration of one experimental set on 30th September 2025, indicating that temperature varied within ±2 ˚C.

Time (min)	Temperature (˚C)
0	25.0
10	25.3
20	25.3
30	25.3
40	25.2
50	25.2
60	25.1
70	24.8
75	24.8
80	24.8
90	24.8
100	24.7
110	24.7
115	24.7
120	24.7
125	24.7
130	24.7
140	24.7

S6. Predictions of Sugar Compositions in Ternary Mixture

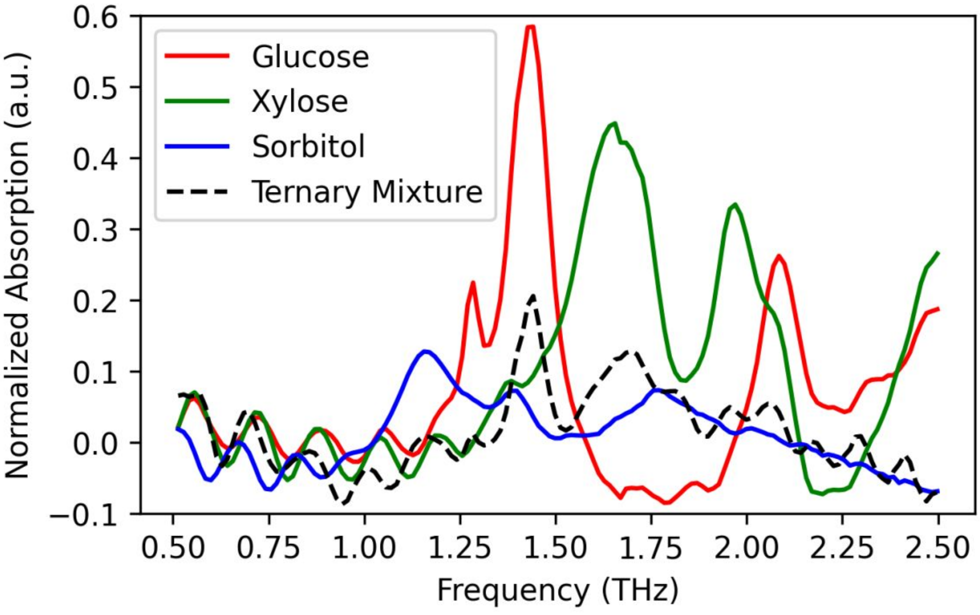

Figure S3. Average post-processed spectra of pure glucose, xylose, and sorbitol (33:33:34% w/w glucose, xylose, and sorbitol) collected over 3 different pellets (n = 30 for each type) and average post-processed spectrum of ternary mixture collected over 4 different pellets (n = 40). All samples were prepared a day prior to their first spectral measurements.

Figure S3 presents the average spectra of pure glucose, xylose, and sorbitol pellets (33:33:34% w/w glucose, xylose, and sorbitol), each averaged over three different samples (n = 30 per type), as well as the average spectrum of the ternary mixture obtained from four different samples (n = 40). Note that all spectra were post-processed according to the same procedures described in Section S2. As shown in the figure, the average spectrum of the ternary mixture exhibits features that resemble a combination of the spectra of the pure components. However, it does not closely match most of the binary mixture spectra shown in Figure 2. Note

that while spectrum of the ternary mixture appears the most similar to that of the binary mixture containing equal proportions of glucose and fructose, this similarity could potentially lead to misinterpretation by the machine learning model trained on binary mixture datasets. Table S5 summarizes the RMSE values of the SVR (best-performing) models when predicting the sugar composition of the ternary mixture using SVR models trained on binary mixture datasets. As expected, the RMSE values are higher than those reported in Section 3.2.

Table S5. Average predicted concentration and RMSE value determined for each sugar type in the ternary mixture (glucose & xylose & sorbitol) using the SVR model trained from binary mixture datasets. Those metrics were calculated from 40 test spectra (4 samples per experiment × 10 test spectra per sample) collected within 1.0 – 2.5 THz frequency range.

Dataset	Predicted Concentration [RMSE] (% w/w)		
	Glucose	Xylose	Sorbitol
Glucose & Sorbitol	48.80 [16.00]	N/A	51.20 [17.39]
Xylose & Xylitol	N/A	30.04 [3.06]	N/A
Glucose & Fructose	54.02 [21.03]	N/A	N/A
